# Risk factors for respiratory *Aspergillus fumigatus* in German Cystic Fibrosis patients and impact on lung function

**DOI:** 10.1038/s41598-020-75886-w

**Published:** 2020-11-04

**Authors:** Uta Düesberg, Julia Wosniok, Lutz Naehrlich, Patience Eschenhagen, Carsten Schwarz

**Affiliations:** 1 Mukoviszidose Institute, In den Dauen 6, 53117 Bonn, Germany; 2 Interdisciplinary Center Clinical Trials (IZKS), University Medical Center Mainz, Langenbeckstraße 1, Mainz, Germany; 3 Department of Pediatrics, Justus-Liebig-University Giessen, Feulgenstr. 10-12, 35392 Giessen, Germany; 4 Department of Pediatric Pneumology, Immunology and Intensive Care Medicine, Cystic Fibrosis Center, Charité – Universitätsmedizin Berlin, Augustenburger Platz 1, 13353 Berlin, Germany

**Keywords:** Respiratory tract diseases, Cystic fibrosis

## Abstract

Airway inflammation and chronic lung infections in cystic fibrosis (CF) patients are mostly caused by bacteria, e.g. *Pseudomonas aeruginosa* (PA). The role of fungi in the CF lung is still not well elucidated, but evidence for a harmful and complex role is getting stronger. The most common filamentous fungus in CF is *Aspergillus fumigatus* (AF). Age and continuous antibiotic treatment have been discussed as risk factors for AF colonisation but did not differentiate between transient and persistent AF colonisation. Also, the impact of co-colonisation of PA and AF on lung function is still under investigation. Data from patients with CF registered in the German Cystic Fibrosis Registry database in 2016 and 2017 were retrospectively analysed, involving descriptive and multivariate analysis to assess risk factors for transient or persistent AF colonisation. Age represented an independent risk factor for persistent AF colonisation. Prevalence was low in children less than ten years, highest in the middle age and getting lower in higher age (≥ 50 years). Continuous antibiotic lung treatment was significantly associated with AF prevalence in all age groups. CF patients with chronic PA infection had a lower lung function (FEV1%predicted), which was not influenced by an additional AF colonisation. AF colonisation without chronic PA infection, however, was significantly associated with a lower function, too. Older age up to 49 years and continuous antibiotic use were found to be the main risk factors for AF permanent colonisation. AF might be associated with decrease of lung function if not disguised by chronic PA infection.

## Introduction

Airway inflammation and chronic lung infections are the leading cause of morbidity and mortality in cystic fibrosis (CF) patients. Lung infections are mostly caused by bacteria. The most common bacterial infective agent in CF patients is *Pseudomonas aeruginosa* (PA) which is known to have an impact on decline lung function and to increase mortality ^1,2^. PA prevalence in German CF patients is 40.7% in the whole CF registry population, with rising numbers at an older age (19.8% in patients younger than 18 years and 57.3% in adult patients)^3^. Despite treatment options of initial PA infection, permanent elimination is not always possible, and chronic infection is common in older patients (53.1% in adult patients vs. 9.8% in patients younger than 18 years)^3^, with accordingly long-term antibiotic use.

Microbiologic diagnostic and treatment so far concentrate mainly on bacterial infections but the ongoing investigation of the complex lung micro- and mycobiome also shows the relevance of fungi^4^. Nevertheless, the pathological and clinical role of fungi in the CF lung, is still not well elucidated. However, evidence for a harmful and complex role of fungi in CF lung disease is getting stronger^5–9^.

Fungal colonisation of the lung is dominated by the yeast *Candida albicans* (CA) and the filamentous fungus *Aspergillus fumigatus* (AF), with a wide-range prevalence of 38–75% and 5–54%, respectively^10–16^. The clinical relevance of *Candida* spp. is not clarified yet, and is controversially discussed. AF, however, is known to be able to cause allergic bronchopulmonary aspergillosis (ABPA) and acute infection of the lung parenchyma (*Aspergillus pneumonia*), which can be severe and difficult to treat^6,7,17^. An additional AF entity is the *Aspergillus bronchitis*^8,18,19^. Several risk factors for AF colonisation, like continuous antibiotic therapy or chronic lung inflammation as well as older age, are currently discussed^20^. One risk factor for ABPA can be pet ownership^21^.

Even intermittent co-colonisation of PA and AF seem to lead to higher hospitalisation and lower lung function in the same extent as chronic PA infection^22,23^. There is evidence, that co-colonisation of PA with fungi leads to an interaction of the species, resulting in changes in microbiome composition. However, it is still under investigation, if coexisting of PA and AF results in antagonistic influence or synergistic effects on the lung inflammation^24–28^.

To increase the evidence about AF impact on CF patients` lung disease, we analysed data from the German CF Registry, and correlated them to lung function, chronic PA infection and treatment for CF lung disease.

## Methods

### Study population

Data from patients with CF registered in the German Cystic Fibrosis Registry database in 2016 and 2017 were retrospectively analysed, including patients from 91 CF centres in Germany and one in Innsbruck, Austria (data status 29.08.2019). In addition to an annual dataset for all patients (group 1) encounter data collection is available for a subset of patients (group 2 and 3). The three differently defined groups are characterised as follows:

- Group 1: All patients of the German CF registry 2017 with no lung transplantation in medical history (n = 5,665) (Annual dataset).
- Group 2: Patients with at least two visits and microbiological diagnostics a year in 2016 (n = 3,698) or in 2017 (n = 4,153). Patients were analysed in three categories: patients with no AF positive culture in respiratory sample, one AF positive culture or at least two AF positive cultures per year, defined as no AF colonisation, transient colonisation and persistent colonisation. For children, BMI was calculated as percentile^29^ (Encounter dataset).
- Group 3: Patients with at least two visits and microbiological diagnostics a year in 2016 (n = 2,529) and 2017 (n = 2,823), who were documented with no AF positive culture or at least two AF positive cultures per year in respiratory sample, and who were older than three years, with no antifungal treatment and no transplantation in medical history. Patients with only one AF positive culture documented in 12 months, were not analysed to exclude patients with a probable transient colonisation of AF. BMI was calculated as percentile for children^29^ and adults as well^30^. (Encounter dataset).

The underlying diagnostic method of AF culture was not determined.

The registry and all its protocols were approved by the German Ethics Committee in Giessen by number AZ24/19. All methods were performed in accordance with relevant guidelines and regulations. Informed consent for data sampling was obtained from all subjects or, if subjects are under 18 years old, from a parent and/or legal guardian.

### Statistical analysis

Group 2 was analysed descriptively and compared by exploratory chi-squared tests or Mann–Whitney U tests at a significance level of alpha = 0.05. To determine risk factors for persistent AF colonisation multivariate analysis was used in group 3. The variables age, gender, best FEV1 (% predicted), BMI percentile, chronic PA infection, genotype, continuous antibiotic therapy, number of exacerbations per year, pancreatic insufficiency, arthritis/arthropathy, diabetes, nasal polyps, ABPA, use of inhaled or oral steroids, use of CFTR modulators and use of azithromycin were pre-selected and analysed by binary logistic regression. Odds ratios and 95% confidence intervals were calculated. All analyses were performed in SAS 9.4 (Statistical Analysis System, SAS Institute Inc., Cary, NC, USA).

### Statement

All experiments were performed in accordance with relevant guidelines and regulations.

## Results

### Prevalence of fungi in CF patients

**Figure 1:**
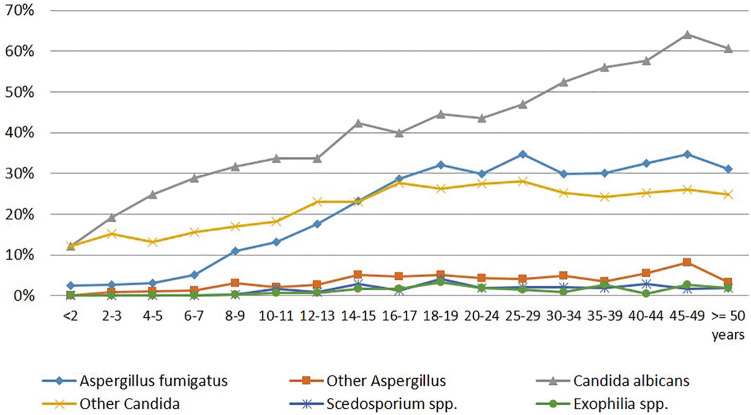
Prevalence of fungi in the 2017 CF registry cohort (n = 5,665) (group 1).

Fungi were present in CF patients of all ages. Especially *Candida* spp. were diagnosed even in young children, but with higher age, colonisation with AF was increasing as well. In patients older than 50 years (group 1), *Candida* spp. and *Aspergillus* spp. prevalence declined (Fig. 1). The most common fungi in CF airways in group 2 was *Candida albicans* (42.8% in 2016 and 42.0% in 2017) and other *Candida* spp. (24.8% and 25.6%), followed by AF (27.3% and 25.5%), other *Aspergillus* spp. (4.8% and 4.6%), *Exophiala* spp. (1.7% and 1.4%) and *Scedosporium* spp.( *Scedosporium apiospermum complex* and *Lomentospora prolificans;* 1.6% and 1.9%).

### Comorbidities in patients with AF

Of the CF patients of group 2 with at least two visits in 2016 and 2017, respectively, 27.3% in 2016 and 25.5% in 2017 had AF diagnosis. In 2016, 15.4% of patients had two or more AF positive cultures and 13.9% in 2017. Demographic data and disease status are shown in Table 1.

**Table 1:** Demographics, disease status, continuous antibiotic treatment and other therapeutics, and bacterial colonisation of CF patients with and without AF diagnosis in group 2. Patients with unknown AF diagnosis are not shown. *Chronic Pseudomonas infection defined by modified Leeds criteria^31^.

	2016					2017				
	No AF diagnosis (n = 2,591)	One positive AF (n = 439)	At least two positive AF (n = 571)		P value no AF vs. one AF one AF vs. ≥ two AF no AF vs. ≥ two AF	No AF diagnosis (n = 2,999)		One positive AF (n = 481)	At least two positive AF (n = 578)	P value no AF vs. one AF one AF vs. ≥ two AF no AF vs. ≥ two AF
Median age (years, range)	16.0 (0.0–78.8)	22.0 (1.0–73.0)	25.0 (3.0–74.0)		< .0001 0.0003 < .0001	16.0 (0.0–79.0)		23.0 (0.0–70.0)	25.0 (6.0–75.0)	< .0001 0.0013 < .0001
Male (%)	53.4	50.8	47.3		0.3166 0.2684 0.0083	52.3		54.9	50.4	0.2826 0.1408 0.4013
Median BMI percentile (range): children	32.0 (15.0–56.0)	24.0 (11.0–46.0)	27.0 (10.0–44.0)		0.0017 0.8511 0.0061	33.0 (15.0–56.0)		26.0 (10.0–48.0)	26.0 (10.0–46.0)	0.0183 0.7625 0.0109
Median BMI (range): adults	21.1 (2.5–46.2)	21.4 (13.9–34.6)	20.9 (12.4- 30.8)		0.9722 0.1298 0.0417	21.1 (13.3–46.1)		20.8 (15.0–37.9)	21.0 (13.6–32.0)	0.3455 0.5051 0.0356
Genotype F508del homozygote (%) F508del heterozygote (%) Other genotype (%)	41.8 35.5 22.6	50.3 33.0 16.6	48.9 34.5 16.7		0.0014 0.8790 0.0014	42.5 35.8 21.7		47.3 37.9 14.8	47.7 35.4 17.0	0.0021 0.5309 0.0165
Pancreatic insufficiency (%)	95.9	95.4	96.0		0.6520 0.6805 0.9450	95.2		96.5	96.2	0.2088 0.8150 0.2831
ABPA (%)	5.8	8.0	9.8		0.0714 0.3127 0.0004	6.2		12.3	9.2	0.0714 0.3127 0.0004
CFRD (%)	16.4	20.3	24.9		0.0714 0.3127 0.0004	17.2		20.6	27.0	0.0714 0.3127 0.0004
Liver disease (%)	28.8	38.7	41.9		< .0001 0.3148 < .0001	26.4		35.1	38.2	< .0001 0.2977 < .0001
Arthritis and/or arthropathy (%)	3.9	4.6	6.3		0.4891 0.2286 0.0091	3.3		5.0	6.8	0.0691 0.2286 0.0001
**Therapy (%)**										
Continuous antibiotics	53.3	71.5	79.0		< .0001 0.0061 < .0001	54.0		72.8	81.5	< .0001 0.0007 < .0001
Tobramycin (inhalative)	18.6	26.0	29.8		0.0003 0.1825 < .0001	18.5		26.2	31.0	< .0001 0.0876 < .0001
Colistin (inhalative)	23.4	33.5	40.3		< .0001 0.0269 < .0001	22.1		39.1	41.7	< .0001 0.3889 < .0001
Aztreonam (inhalative)	11.5	21.2	24.5		< .0001 0.2125 < .0001	12.4		20.2	23.9	< .0001 0.1481 < .0001
Bronchodilatators (inhalative)	82.4	88.4	93.9		0.0020 0.0020 < .0001	82.9		91.9	94.5	< .0001 0.0957 < .0001
Steroids	46.0	57.6	64.6		< .0001 0.0235 < .0001	46.4		54.3	63.5	0.0014 0.0023 < .0001
Inhalative steroids	33.0	45.8	51.5		< .0001 0.0723 < .0001	33.4		43.7	52.4	< .0001 0.0045 < .0001
Oral steroids	8.1	11.6	13.0		0.0140 0.5207 0.0002	8.9		9.6	12.3	0.6384 0.1597 0.0110
Antimycotics	10.5	13.2	12.1		0.0966 0.5921 0.2811	11.1		12.9	13.7	0.2611 0.7106 0.0813
CFTR modulator ivacaftor	3.1	2.1	2.8		0.2338 0.4458 0.7189	3.3		4.0	2.9	0.4409 0.3670 0.6836
CFTR modulator lumacaftor	4.6	10.7	14.2		< .0001 0.0994 < .0001	8.5		12.5	16.8	0.0048 0.0495 < .0001
**Bacterial co-colonization with** ***Pseudomonas aeruginosa, Staphylococcus aureus* and non-tuberculous mycobacteria (%)**										
*Pseudomonas aeruginosa* (PA)*	36.7	52.2	62.2		< .0001 0.0014 < .0001	36.5		54.3	63.5	< .0001 0.0023 < .0001
Mucoid PA	20.5	29.2	30.7		< .0001 0.6083 < .0001	19.8		31.4	35.5	< .0001 0.1623 < .0001
multiresistant PA	11.7	16.2	21.2		0.0090 0.0439 < .0001	12.9		16.8	20.4	0.0188 0.1381 < .0001
*Staphylococcus aureus* (SA)	65.7	69.0	67.6		0.1725 0.6310 0.3826	64.8		70.5	68.9	0.0148 0.5683 0.0596
Non-tuberculous mycobacteria	1.2	3.2	8.8		0.0010 0.0003 < .0001	1.9		6.7	9.0	< .0001 0.1599 < .0001

Nutritional status measured by Body Mass Index (BMI) percentile differed in both years significantly between younger CF patients (< 18 years of age, p < 0.05) with no or with at least two positive AF cultures. In adult patients, there was less difference in BMI (Table 1).

In the group 2 population, 6.7% of patients in 2016 and 7.4% in 2017 suffered from ABPA. Of patients with at least two positive AF cultures, prevalence of ABPA was significantly higher compared to patients with no AF diagnosis (p < 0.05). In 2017, patients with only one AF diagnosis had the highest ABPA prevalence.

The prevalence of CF-related diabetes (CFRD) was significantly higher in patients with at least two positive AF cultures (p < 0.0001), and also liver disease was significantly more common in patients with at least two positive AF cultures in comparison to patients with no AF positive cultures (p < 0.0001). Liver cirrhosis, however, was not more prevalent in any patient group.

Arthritis and/or arthropathy were significantly more common amongst patients with at least two positive AF cultures (p < 0.05).

### Lung function in patients with AF and with or without chronic PA infection

**Figure 2:**
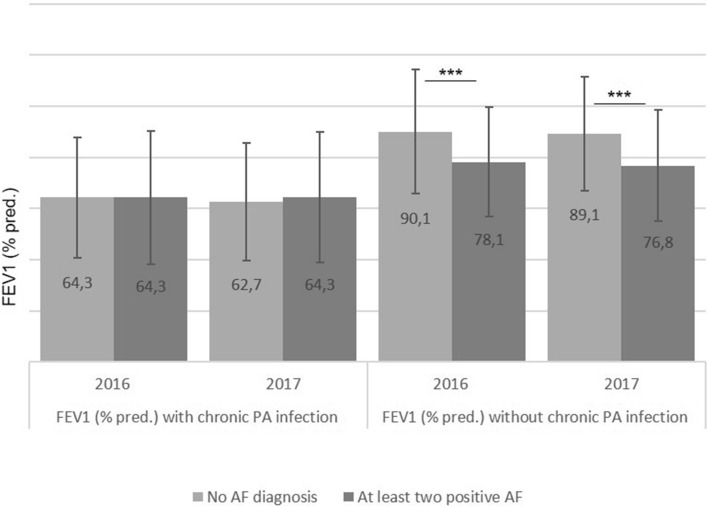
Lung function in CF patients with and without chronic PA infection and no or at least two positive AF cultures (***p < 0.0001) (group 2).

CF patients with chronic PA infection had a significant lower FEV1% pred. than those without chronic PA infection (p < 0.0001). CF patients without chronic PA infection, however, had a significant lower FEV_1_ in case of one or at least two positive AF cultures (p < 0.0001, Fig. 2).

Pulmonary exacerbations requiring antibiotic treatment were experienced by significant more CF patients with at least two positive AF cultures (53.9% vs. 39.5% with no AF diagnosis in 2016 and 73.9% vs. 59.2% in 2017; p < 0.0001, respectively).

### Antibiotic treatment

Continuous antibiotic lung treatment was significantly associated with AF prevalence in children as well as adult patients in 2016 and 2017 (p < 0.0001), see Table 1.

### Bronchodilators and steroids

Most of the patients received continuous bronchodilators. Significantly more patients with at least two positive AF cultures received bronchodilators (p < 0.0001), but also patients with only one AF positive culture (p < 0.005), see Table 1.

About half of the CF population received continuous steroid treatment in 2016 and in 2017, mostly inhalative (37.5% and 37.6%) and rarely oral (9.2% and 9.6%). Significantly more patients with one or more positive AF cultures received steroids (p < 0.0001), see Table 1.

### Antifungal treatment

About ten percent of patients with no documented AF diagnosis received antifungal treatment. Only slightly more patients received this treatment when diagnosed with one or at least two positive AF cultures (Table 1). Itraconazole was the most common antifungal substance prescribed in all patients (7.6% in 2016 and 8.8% in 2017), followed by voriconazole (2.0% and 1.4%), posaconazole (0.9% and 1.0%) as well as amphotericin B (0.8% and 0.9%).

### CFTR modulator therapy

Treatment with ivacaftor was not different in patients with or without AF diagnosis in both years. Prescription of lumacaftor/ivacaftor was higher in 2017 than in 2016 in all patients. Significant more patients with at least two positive AF cultures were treated with lumacaftor than patients with no AF diagnosis (p < 0.0001), see Table 1.

### Bacterial co-colonisation with Pseudomonas, Staphylococcus and Mycobacteria

In patients with at least two positive AF cultures, significantly more PA, as well as mucoid PA and multiresistant PA were diagnosed compared to patients with no AF diagnosis in both years (p < 0.0001, Table 1). Prevalence of *Staphylococcus aureus* (SA), in contrast, was not significantly different in the three AF groups (Table 1). Non-tuberculous mycobacteria were diagnosed significantly more in patients with at least two positive AF culture (p < 0.0001, Table 1). The most common non-tuberculous mycobacteria were *Mycobacteria abscessus* (5.3% in 2016 and 5.4% in 2017 vs. 0.7% and 1.2% in patients with no AF positive culture).

### Risk factors for persistent AF colonisation

In order to evaluate risk factors for persistent AF colonisation, a multivariate analysis was performed with patients showing no AF or at least two positive AF cultures (group 3, see Methods), reflecting a persistent AF colonisation. Patients with only one positive AF culture in 12 months were excluded to rule out transient AF colonisation. Median values and proportions of variables analysed in group 3 did not vary widely from those of group 2.

**Figure 3:**
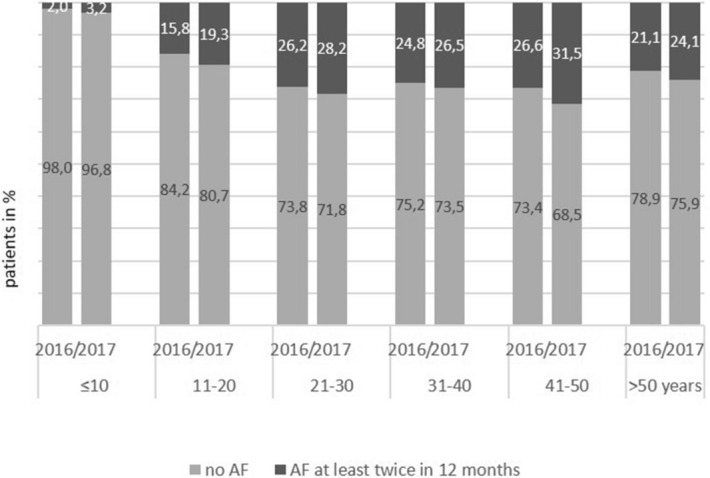
Number of patients with AF positive culture twice in 12 months or no AF positive culture in age categories (group 3).

Age represented an independent risk factor for AF positive cultures at least twice in 12 months (p < 0.0001). Prevalence was low in children less than ten years, highest in the middle age and getting lower in higher age (Fig. 3). Accordingly, odds ratios as presented in Table 2 indicate an 8.42 to 11.24-fold higher risk for persistent AF colonisation in the middle age compared to children less than 10 years**.**

**Table 2:** Odds ratios and corresponding 95% Wald CI for variables with statistically significant p-values in the multivariate analyses.

2016			2017		
	Odds ratio	95% Wald CI		Odds ratio	95% Wald CI
Age group 21–30 years*	8.42	5.10–13.88	Age group 21–30 years*	10.75	6.23–18.57
Age group 31–40 years*	7.90	4.55–13.71	Age group 31–40 years*	9.49	5.25–17.14
Age group 41–50 years*	11.24	6.07–20.83	Age group 41–50 years*	10.56	5.52–20.20
Continuous antibiotic lung treatment**	2.01	1.50–2.70	Continuous antibiotic lung treatment**	2.29	1.71–3.06
CFTR modulators**	1.62	1.20–2.20	Pancreatic insufficiency**	1.51	1.02–2.23
Number of exacerbations	1.25	1.16–1.34	BMI percentile	0.99	0.99–1.00

*Compared to patients aged ≤ 10 years, ** exposed vs. unexposed, CI = Confidence Interval.

The second significant risk factor for AF colonisation at least twice a year was continuous antibiotic treatment (p < 0.0001). Patients with continuous antibiotic treatment had a twice as high risk of AF colonisation than patients without treatment.

High numbers of pulmonary exacerbations were significantly associated with a higher risk for at least two positive AF cultures in 2016 (p < 0.0001), but not in 2017. Pancreatic insufficiency was an inconsistent risk factor, with significant impact in 2017 other than in 2016. Also, CFTR modulator therapy had a significant impact on AF colonisation in 2016, but not in 2017.

## Discussion

This paper represents the first fungi data analysis of the German CF patient registry to evaluate risk factors for AF colonisation. As described in other publications with smaller patient numbers, filamentous fungi were highly prevalent in CF patients with AF found to be the most common^10–15^. AF prevalence, however, might be underestimated. In pulmonary exacerbations, there is a need of immediate antibiotic treatment before performing fungi diagnostics. Moreover, culture-based methods might miss fungi prevalence in the sputa^32^.

Reported prevalence from other publications varies in a wide range. This could be due to geographic distinctions, different treatment regimens with varying intense antibiotic use, and different diagnostic methods. The sensitivity of the diagnostic method is different, as shown from 18% with conventional microbiological culture to 100% with molecular methods^13^. In our registry analysis, the diagnostic method of AF culture was not determined.

In order to describe AF impact on CF patients, we used different models for analysis in three groups. The group from the whole German registry showed, that the prevalence of AF as well as *Candida* spp. increased with patient`s age. These findings correspond with results from other authors^10,14,33^. Our results also show a decrease in AF prevalence in CF patients older than 50 years. This might reflect that getting older with CF presupposes a milder CF disease or a better lung health status, which prevents from AF colonisation. Nevertheless, older age was found to be the strongest risk factor for persistent AF colonisation.

In order to differentiate between transient and persistent AF colonisation, two different AF positive groups were analysed. Patients who were documented at least twice in 12 months were divided in two groups with one and with at least two AF positive cultures a year. One AF positive culture would reflect a transient colonisation with AF, whereas at least two positive AF cultures a year would reflect a persistent AF colonisation or even clinically relevant infection. Therefore, only the group of patients with at least two positive AF cultures a year was used to analyse the risk factors for persistent AF colonisation. Patients with antifungal treatment have been withdrawn from the multivariate analysis to exclude patients with negative AF diagnosis due to effective antifungal treatment. As seen in the descriptive data, about 10% of patients with no AF colonisation received antifungal treatment.

Our findings show a higher prevalence of comorbidities like CFRD, liver disease and arthritis/arthropathy in patients with AF colonisation but were not seen as a risk factor for persistent AF colonisation. Accordingly, bronchodilator and steroid treatment showed no impact on persistent AF colonisation, despite increased use of bronchodilators and steroids in patients with AF colonisation. In addition, bacterial co-colonisation was higher in patients with AF colonisation. Taken into consideration that all these factors might reflect older patients, the multivariate analysis (considering age as an influencing parameter), did not show any of the comorbidities, bronchodilator or steroid therapy, or bacterial co-colonisation as risk factor for persistent AF colonisation. Also, patients with more pulmonary exacerbations had a higher probability for AF colonisation, but this observation was not consistent as a risk factor as well.

Children with one or more AF positive cultures had a higher BMI, whereas adults had a lower one. However, using multivariate analysis, body weight was not a consistent risk factor for persistent AF colonisation.

Lung function is known to decline in patients with chronic PA infection^1,2^. This was seen in the German CF patients as well, irrespective of additional AF colonisation. However, patients with no chronic PA infection nor AF colonisation had a significantly higher lung function than patients with no PA infection but with persistent AF colonisation. This implies a direct impair of lung function by persistent AF colonisation, also reported by Pihet et al.^15^. They assumed that AF metabolites and proteolytic enzymes might damage the lung epithelia directly.

Chronic PA infection itself was not a risk factor for AF colonisation in our analysis but continuous antibiotic treatment (oral and inhalative) was a risk factor for persistent AF colonisation. Antibiotic use was reported as a risk factor for AF colonisation previously^14,20^.

It has been discussed, that CFTR genotype might correlate with infection and pathogenicity of certain pathogens, and interfere with CFTR modulator treatment^34–36^. Descriptive data from our analysis also show a higher proportion of patients with F508del homozygote genotype in patients with AF colonisation and a slightly higher proportion of patients with pancreatic insufficiency. Nevertheless, we did not find a consistent correlation between CFTR genotype or pancreatic insufficiency and persistent AF colonisation. We also did not find a correlation between AF prevalence and ivacaftor therapy, but the number of patients (3%) receiving ivacaftor was low. Significantly more patients with AF diagnosis than without AF diagnosis received therapy with lumacaftor/ivacaftor. Lumacaftor/ivacacftor, however, was only approved for patients older than 12 years in 2016 and 2017, so it could not be analysed as a risk factor for the complete patient cohort.

Our study had some limitations. The study analysed data retrospectively over two years. Therefore, we could not analyse a possible long-term effect of chronic PA. Additionally, the quality of sputum sampling and the method of AF culturing was not standardised. Negative AF culture was assumed, if patients have been documented with negative AF culture as well as with microbiological testing without AF testing. This could contain false positive or false negative AF findings. Furthermore, co-colonisation of *Staphylococcus aureus* as also important pathogen in CF airways was not addressed but could have an impact on AF, too.

Concerning the results, age was the strongest risk factor for AF colonisation in our study. This might reflect an age bias, as patients older than 50 years were colonised less than younger CF patients. The younger patient group is potentially more diverse in health status, with a higher proportion of severely ill patients or a faster declining lung function. These CF patients would not reach the age of 50 years.

In conclusion, in the German CF patient cohort of 2016 and 2017 the risk factors for persistent AF colonisation were age and continuous antibiotic treatment. The persistent AF colonisation in CF patients` lung is associated with low lung function if not co-colonised with PA. However, further studies are needed to elucidate the clinical impact of AF for CF patients and to define patients who would benefit from an antifungal treatment to prevent lung function decline or lung damage.
